# Advances in the Treatment of Cognitive Impairment in Schizophrenia: Targeting NMDA Receptor Pathways

**DOI:** 10.3390/ijms251910668

**Published:** 2024-10-03

**Authors:** Ting Zhang, Chang Liu, Ning Zhong, Yichen Wang, Yiyun Huang, Xiaoqin Zhang

**Affiliations:** Department of Pharmacology, Health Science Center, Ningbo University, Ningbo 315211, China; m15958215021@163.com (T.Z.); changbwel0310@163.com (C.L.); zn1073669134@163.com (N.Z.); m13336629100@163.com (Y.W.); hyy18135@163.com (Y.H.)

**Keywords:** NMDA receptor, cognitive impairment, excitation/inhibition balance, schizophrenia

## Abstract

Cognitive impairment is a core feature of schizophrenia, playing a pivotal role in the pathogenesis and prognosis of this disorder. Cognitive impairment in schizophrenia encompasses a wide range of domains, including processing speed, episodic memory, working memory, and executive function. These deficits persist throughout the course of the illness and significantly impact functional outcomes and quality of life. Therefore, it is imperative to identify the biological basis of cognitive deficits in schizophrenia and develop effective treatments. The role of N-methyl-D-aspartate (NMDA) receptors in synaptic transmission and plasticity has long been recognized, making them potential targets for schizophrenia treatment. This review will focus on emerging pharmacology targeting NMDA receptors, offering strategies for the prevention and treatment of cognitive deficits in schizophrenia.

## 1. Introduction

Schizophrenia is a chronic and severe mental disorder, typically emerging during adolescence or early adulthood, with an estimated lifetime prevalence of approximately 1% [1]. It often manifests with positive symptoms, such as hallucinations and delusions, and negative symptoms characterized by diminished motivation and reduced emotional expression, as well as cognitive impairment, including decreased attention and memory. Importantly, cognitive impairment associated with schizophrenia (CIAS) is highly prevalent, affecting approximately 80% of individuals with the disorder. CIAS often precedes the onset of positive and negative symptoms and persists throughout the course of the illness. Even after achieving “stability” in positive symptoms through the usage of antipsychotic medications, many patients continue to experience cognitive impairment, which in turn contributes to poorer functional outcomes [2,3]. Therefore, CIAS is considered the strongest predictor of long-term functional prognosis in individuals with schizophrenia.

**Figure 1:**
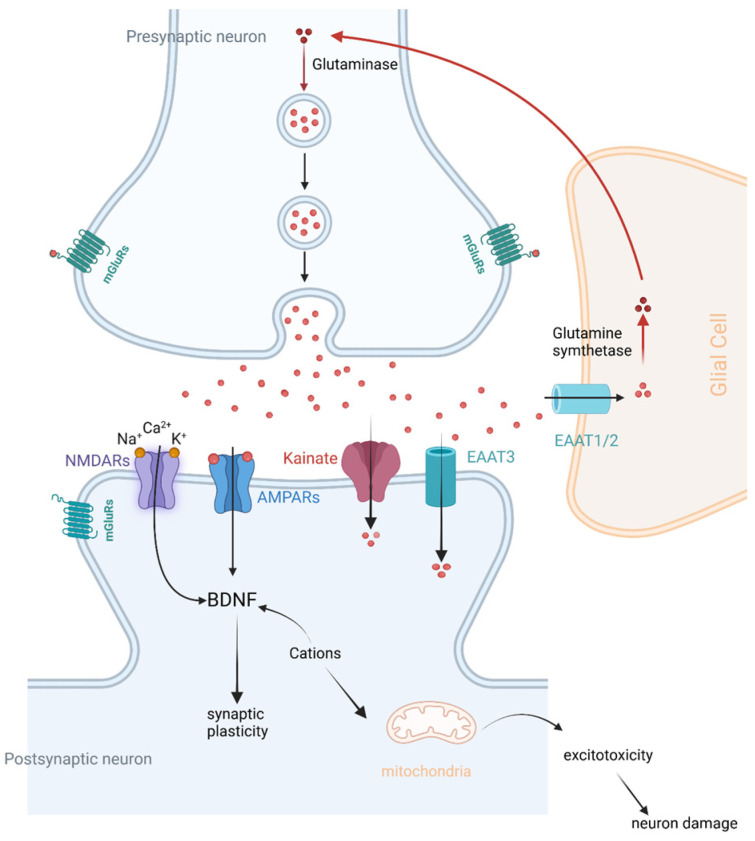
A schematic overview of the glutamatergic system in the brain, including glutamate synthesis and release, and the subsequent glutamate activities through its receptors. EAATs, excitatory amino acid transporters; NMDARs, N-methyl-D-aspartate receptors; AMPARs, α-amino-3-hydroxy-5-methyl-4-isoxazole propionic acid receptors; BDNF, brain-derived neurotrophic factor. Figure created with Biorender.com (accessed on 20 July 2024).

Treating cognitive impairment is a crucial aspect of improving functional outcomes in schizophrenia. The glutamatergic system, illustrated in Figure 1, is one of the key mechanisms underlying cognitive deficits. The glutamatergic system plays a central role in various cognitive processes, including learning, memory, and executive function [4]. Dysfunction within this neurotransmitter system has been implicated in the cognitive impairment observed in schizophrenia. The glutamate hypothesis of schizophrenia is based on the ability of N-methyl-D-aspartate (NMDA) receptor antagonists to induce schizophrenia-like symptoms, as well as emergent literature demonstrating disturbances in NMDA receptor-related gene expression and metabolic pathways in schizophrenia [5]. Therefore, it seems that NMDA receptor and synapse function may participate in the pathogenesis of schizophrenia, suggesting that modulators of NMDA receptor signaling are promising candidates for therapy to treat CIAS.

Neurotrophic factors play a vital role in neuronal development, synaptogenesis, and the response to stress and anxiety, and they have been linked to the pathogenesis of schizophrenia [6,7]. Brain-derived neurotrophic factor (BDNF) is one of the most widely distributed and extensively studied neurotrophins in the brain, crucial for the plastic changes associated with learning and memory [8]. Alterations in BDNF signaling, which regulates synaptic function via tropomyosin-related kinase B receptors (TrkB), are implicated in schizophrenia alongside NMDA receptor hypofunction [9]. Dysfunctional BDNF signaling resulting from NMDA receptor activity deficits may contribute to the cognitive impairment observed in the PCP model [10]. Moreover, patients with an earlier age of onset exhibit more negative symptoms and cognitive deficits, correlating with lower serum BDNF levels [11]. Consequently, reduced BDNF levels at the onset of psychosis indicate its potential role in the pathogenesis of schizophrenia and suggest that it could serve as a valuable neurobiological marker for early treatment interventions targeting NMDA receptor pathways.

## 2. NMDA Receptor Function and Cognition

NMDA receptors are cation-selective ligand-gated ion channels that, together with other ionotropic receptors (AMPA receptors, kainate receptors) and G-protein-coupled receptors (metabotropic glutamate receptors, or mGluRs), mediate glutamatergic synaptic transmission throughout the central nervous system (CNS) [12]. The channels contain two obligatory GluN1 and two modulatory GluN2 (A-D) subunits or a combination of GluN2 and GluN3 subunits. The activation of NMDA receptors is both voltage-dependent and ligand-gated, requiring the binding of two ligands: glutamate and either D-serine or glycine. Glutamate serves as the neurotransmitter, released from presynaptic terminals in an activity-dependent manner, while D-serine or glycine acts as a modulator, maintaining relatively constant levels in the extracellular fluid. The ion-channel integral to the NMDA receptor is voltage-dependently blocked by magnesium ions (Mg^2+^), and depolarization removes this block [13,14,15]. Thus, the NMDA receptor serves as a coincidence detector, linking neurotransmitter activation with the electrical properties of neurons. Sustained NMDA receptor activation triggers signaling to the nucleus and coordinated changes in gene expression, supporting the establishment of long-term synaptic plasticity (LTP), which is the basis of learning and memory [16]. However, excessive glutamate can chronically overstimulate NMDA receptors, resulting in excessive intracellular calcium and excitotoxicity, a mechanism implicated in neuronal death in various CNS disorders. Both a deficiency and an excess of NMDA receptor activity can be detrimental. Excitotoxicity associated with NMDA receptors has prompted the search for antagonists as potential neuroprotective agents, while their role in synaptic plasticity has inspired research into receptor potentiators for treating cognitive dysfunction.

NMDA receptors are integral to the intricate processes of synaptic transmission, neuronal plasticity, and cognitive function in the brain [17]. Their uniqueness among glutamate receptors lies in their high calcium permeability and voltage-dependent activation properties. This distinctiveness allows NMDA receptors to act as molecular switches that mediate critical aspects of synaptic plasticity, particularly LTP [18]. Specifically, activation of NMDA receptors permits the influx of calcium ions into the postsynaptic neuron. This influx of calcium serves as a pivotal signal, triggering a cascade of intracellular events that profoundly impact synaptic strength and neuronal connectivity, including the activation of protein kinases, such as calcium/calmodulin-dependent protein kinase II (CaMKII) and protein kinase C (PKC). The phosphorylation of kinases influences target proteins, leading to changes in synaptic efficacy, including alterations in the function and density of neurotransmitter receptors at the synapse [19]. Furthermore, activation of NMDA receptors during LTP induction also influences gene expression within the neuron. This modulation of gene transcription and translation is mediated by calcium-dependent signaling pathways and transcription factors like cAMP response element-binding protein (CREB). When CREB is activated, it promotes the expression of genes such as BDNF, which is crucial for synaptic strengthening and the structural remodeling of synapses, thereby contributing to the consolidation of memory [20].

## 3. NMDA Receptor Hypofunction in Schizophrenia

The normal functioning of NMDA receptors is crucial for maintaining cognitive functions such as learning and memory. The dysfunction of NMDA receptors is a key mechanism underlying schizophrenia, especially CIAS [21,22]. Early findings have shown that NMDA receptor antagonists, such as phencyclidine (PCP), ketamine, and MK801, can induce schizophrenia-like behaviors, including cognitive impairment, in both preclinical and clinical studies [23,24]. Animal studies have confirmed that administering NMDA receptor antagonists like MK-801 leads to cognitive impairment across multiple domains in rodents [25]. NMDA receptor subunit NR1 knockdown mice exhibit behavioral abnormalities, which can be ameliorated to varying degrees with antipsychotics or psychoactive drugs [26,27,28]. Furthermore, autoimmune encephalitis linked to NMDA receptor-specific antibodies has been associated with severe psychosis [29]. Inflammation and elevated levels of pro-inflammatory cytokines in individuals with psychotic disorders can activate tryptophan metabolism through the kynurenine pathway, resulting in the overproduction of kynurenic acid, an endogenous allosteric antagonist of NMDA receptors [30,31,32]. Increased kynurenine and kynurenic acid levels have been detected in the cerebrospinal fluid (CSF) [33,34,35] and blood [36,37] of patients with schizophrenia, which antagonize the effects of glycine at the glycine site of the NMDA receptor and contribute to CIAS [36,38,39,40]. Interestingly, kynurenic acid levels are lower in female Caucasians, which may help explain their lower incidence of schizophrenia [41]. Collectively, both genetic and environmental factors that precipitate NMDA receptor hypofunction have been implicated in the disease progression and symptoms of schizophrenia [42]. Specifically, NMDA receptors can affect cognitive function at the cellular and neural network levels.

### 3.1. Cellular and Molecular Level: Synaptic Plasticity

Synaptic plasticity is the experience-dependent change in connectivity between neurons that is believed to underlie learning and memory [43]. Patients with schizophrenia who experienced negative life events, such as uterine infection or pregnancy complications, psychosocial issues, amphetamine abuse, autoimmune disease, and other brain traumas, can experience activity-dependent modifications to NMDA receptor function that are essential to synaptic transmission and the establishment of neuronal circuits. Postmortem studies of the brains of patients with schizophrenia have revealed lower levels of expression of the obligatory NMDA receptor subunit GluN1 [44], increased expression in the endogenous NMDA receptor antagonist kynurenate [32], and a reduction in levels of the NMDA receptor co-agonist D-serine, along with reduced activity of its synthesizing enzyme, D-serine racemase [45]. Additionally, biological pathway analyses of genome-wide association studies’ (GWAS) data (~60,000 subjects from the Psychiatric Genetics Consortium) revealed that genetic variants associated with schizophrenia are enriched in pathways related to the postsynaptic density, the postsynaptic membrane, dendritic spines, and histone methylation [46]. Analyses of copy number variants (CNV) have linked de novo mutations in genes encoding the NMDA receptor and proteins that are associated with postsynaptic density to a higher risk of schizophrenia [46,47].

Changes in NMDA receptor expression and function can enhance or suppress synaptic transmission efficacy. For instance, LTP facilitates memory formation, long-term depression (LTD), verifies memory content, and maintains a balance between memory and forgetting. Accordingly, NMDA receptor hypofunction has been linked to various behavioral manifestations of schizophrenia, including social withdrawal, increased locomotor activity, and cognitive impairment, as observed in both humans and animal models [48,49,50]. Our findings revealed that diminished excitatory neurotransmission in the medial prefrontal cortex could be a common pathophysiology, regardless of the prenatal and postnatal pathogenesis in developmental models of schizophrenia, that might underlie the mechanism of defective working memory in those models [51]. Activation of neuregulin 1, a growth factor genetically linked to schizophrenia in humans [52], promotes the internalization of NMDA receptors from the cell surface by an actin-dependent mechanism in prefrontal pyramidal neurons [53]. Additionally, overactivation of the ErbB4 receptor by neuregulin suppresses NMDA receptor activity in the prefrontal cortex of patients with schizophrenia [54], eliciting schizophrenic-like symptoms.

### 3.2. Neural Network Level: Excitation/Inhibition Balance and Neural Oscillation

**Figure 2:**
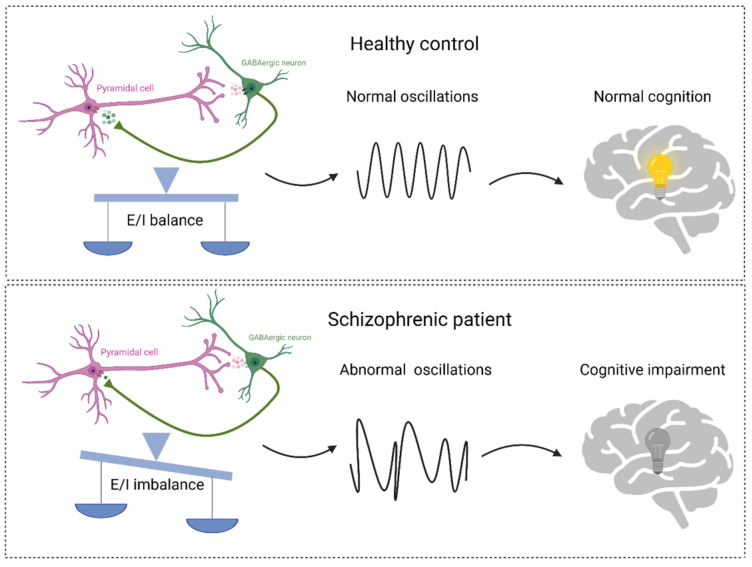
The balance between excitation (E) and inhibition (I), as well as neural oscillations, form the foundation of cognitive function. In healthy individuals, a well-regulated E/I neural network sustains normal oscillations and cognitive function. However, E/I imbalance leads to abnormal oscillations, consequently resulting in cognitive impairment. Figure created with Biorender.com (accessed on 20 July 2024).

Excitatory synaptic transmission and inhibitory synaptic transmission are the two main synaptic transmissions in our brain [55]. The balance between excitatory and inhibitory synaptic transmission (E/I balance) is essential for normal neural development, behavior, and cognition, whereas an E/I imbalance leads to neurological disorders, such as schizophrenia [56]. Importantly, NMDA receptors regulate α-amino-3-hydroxy-5-methylisoxazole-4-propionate receptor (AMPAR)-mediated excitatory and γ-amino- butyric acid receptor (GABAR)-mediated inhibitory synaptic transmission, suggesting that NMDA receptors play an important role in the establishment and maintenance of the E/I balance [57]. Hypofunction in NMDA receptors have been found in human and animal models of schizophrenia, which can impact the balance between excitation (E) and inhibition (I) [22,58,59]. Specifically, within inhibitory neurons, reduced NMDA receptor function leads to a decrease in GABA neurotransmission. Consequently, excitatory neuron inhibition is relieved, leading to an increase in excitatory neurotransmission. This imbalance in E/I results in abnormal neural oscillations and cognitive impairment (Figure 2) [60,61].

Neural oscillations are a crucial mechanism for establishing precise temporal coordination between neuronal responses, which are highly relevant for cognitive processes like memory, perception, and consciousness. In patients with schizophrenia, the synchronization of gamma-band activity is abnormal, suggesting the crucial role of dysfunctional oscillations in generating the cognitive deficits and other symptoms of this disorder. The neurocircuitry hypothesis may help explain how reduced NMDAR activity contributes to the symptoms of schizophrenia [62]. One hypothesis is that cognitive impairment may arise from hypofunctional NMDA receptors on cortical GABA interneurons, particularly fast-spiking parvalbumin interneurons, leading to changes in cortical network oscillations [63,64]. This hypothesis is supported by the fact that patients with schizophrenia have reduced parvalbumin expression in the dorsolateral prefrontal cortex (PFC) and abnormal gamma-band oscillations [63,65], which have been implicated in the synchronization of neural ensembles during working memory and attention. Moreover, NMDA receptor hypofunction on cortical interneurons could boost glutamatergic projection neurons’ activity excessively, leading to the hyperstimulation of GABAergic interneurons in the ventral tegmental area. This overstimulation can dampen the meso-cortical dopamine pathway, causing insufficient dopamine release in the PFC and potentially contributing to cognitive and negative symptoms [66]. In individuals with schizophrenia, there is an upregulation of the serotonin (5-HT) receptor subtype 5-HT1A and a downregulation of 5-HT2A in the PFC [67], which are important for emotion and cognition and involved in modulating NMDA receptor activity [68,69]. Consequently, alterations in 5-HT signaling could potentially influence cognitive and negative symptoms by impacting NMDA receptor function in the PFC.

Overall, NMDA receptors are critical for synaptic plasticity, learning, and memory processes in the brain. They play a crucial role in regulating the balance between excitatory and inhibitory neurotransmission. Dysfunction in NMDA receptor signaling disrupts this balance, leading to the aberrant neural circuitry function and cognitive deficits observed in schizophrenia.

## 4. Targeting NMDA Receptors in Schizophrenia

**Figure 3:**
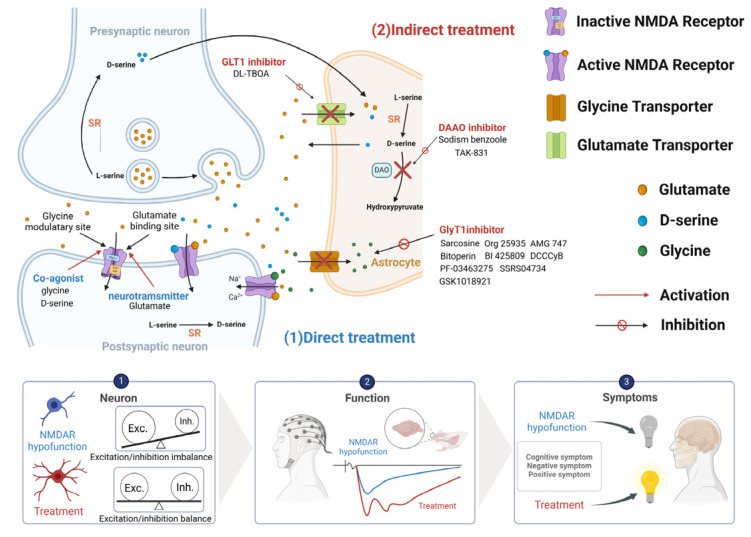
An overview of direct/indirect treatments in the regulation of NMDA receptor function, and the therapeutic effects and possible underlying mechanisms in the treatment of schizophrenia. (1) Enhancing NMDA receptor function through direct treatments (blue: e.g., glutamate, glycine, and D-serine). (2) Boosting NMDA receptor function via indirect treatments (red: e.g., GLT1 inhibitors, DAAO inhibitors, and GlyT1 inhibitors). Bottom panels: the effects of NMDA receptor activation on neural morphology and brain activity, which contribute to the amelioration of symptoms in schizophrenia. GLT1, glutamate transporter; GlyT1, glycine transporter; SR, serine racemase; DAAO, D-amino acid oxidase. Figure created with Biorender.com (accessed on 20 July 2024).

Given the critical role of NMDA receptors in cognition and their implication in schizophrenia, targeting NMDA receptors presents a promising approach for treating CIAS. Notably, strategies targeting the glycine binding site are expected to have less adverse effects compared to modulating the glutamate binding site [70]. Approaches to enhance NMDA receptor function directly or indirectly include modulating glycine or D-serine concentrations in the synaptic cleft and developing selective NMDA receptor modulators (Figure 3).

### 4.1. Direct Enhancement of NMDA Receptor Function

A wide range of compounds that directly activate NMDA receptors, such as glycine and D-serine, have been shown to be effective for improving cognitive function and reducing symptom severity in schizophrenia patients in clinical trials. These agonists act by enhancing NMDA receptor-mediated neurotransmission, thereby restoring synaptic plasticity and neural circuitry function.

#### 4.1.1. Enhancement of NMDA Receptor Functions by Glycine

Glycine is the simplest amino acid and acts as a neurotransmitter in the brain. When glycine receptors are activated, chloride enters the neuron via ionotropic receptors, causing an inhibitory postsynaptic potential. Glycine is also a required co-agonist, along with glutamate, for NMDA receptors. In contrast to the inhibitory role of glycine in the spinal cord, this behavior is facilitated at the NMDA receptors, which are excitatory [71]. Postmortem studies of schizophrenic patients have revealed increases in NMDA-associated glycine binding sites in the cerebral cortex [72]. High serum glycine levels have been reported in chronic schizophrenia patients with pre-pulse inhibition deficits [73]. Similarly, rats treated with a glycine-rich diet display disturbances in sensory gating [74]. Conversely, some studies have reported lower plasma glycine levels in schizophrenia patients compared to healthy controls, and these lower glycine levels were associated with a greater severity of negative symptoms [75]. These findings suggest that glycine levels may compensate for changes in glutamate NMDA receptor transmission in patients with schizophrenia. Further large-scale studies measuring glycine concentrations in both serum and cerebrospinal fluid are needed to elucidate the complex relationship between glycine signaling and the pathogenesis of schizophrenia.

Although there is controversy over glycine levels in patients with schizophrenia, glycine-induced NMDA receptor-mediated enhanced neurotransmission is considered a potentially safe and feasible method for improving negative symptoms as well as CIAS. It has been reported that the potentiation of NMDA receptor function by the infusion of glycine into the prefrontal cortex ameliorated PCP-induced behavioral deficits in latent learning [76]. Glycinamide, a prodrug of glycine, can be converted to glycine in the CNS, and it prevented MK-801-induced deficits in a novel object recognition task in rabbits [77]. Briefly, Table 1 summarizes the key findings from several clinical studies examining the effects of glycine supplementation on the treatment of schizophrenic symptoms. The studies varied in sample size and glycine dosage, but the majority reported significant improvements in negative symptoms and cognitive deficits of schizophrenia with glycine treatment. However, several studies did not find any significant effects on symptoms. The lack of consistency across trials may be due to small sample sizes, various doses of glycine, different trial durations, and clinical ratings. Overall, the evidence suggests that glycine may be a promising adjunctive therapy for targeting the cognitive and negative symptom domains in schizophrenia.

#### 4.1.2. Enhancement of NMDA Receptor Function by D-Serine

D-serine is an endogenous ligand for the glycine site of the NMDA receptor [93]. For the GluN1/N2 subunits of the NMDA receptor, the binding affinity of D-serine is three-fold more potent than that of glycine [94]. Notably, D-serine is the primary co-agonist of synaptic NMDA receptors, whereas glycine is the primary co-agonist of extrasynaptic NMDA receptors [95]. The differential localization and co-agonist preferences of synaptic versus extrasynaptic NMDA receptors have important implications for their distinct roles in neuronal signaling and synaptic function. Synaptic NMDA receptors are typically associated with excitatory neurotransmission and the induction of LTP, processes critical for learning and memory. Moreover, depletion of D-serine diminishes NMDA receptor activity [93] and LTP [96]. In contrast, extrasynaptic NMDA receptors have been linked to excitotoxicity and the propagation of pathological signals, which may contribute to the cognitive deficits observed in neuropsychiatric disorders such as schizophrenia [97,98].

Accumulating evidence highlights the potential therapeutic role of D-serine in the modulation of NMDA receptor function for the treatment of schizophrenia. Indeed, reduced levels of D-serine have been found in the serum of patients with schizophrenia compared to healthy individuals [99]. A postmortem brain study also revealed decreased D-serine in the CSF of schizophrenia patients [100]. Correspondingly, D-serine supplementation during juvenile and adolescent stages has been shown to prevent the onset of cognitive deficits, as well as the prodromal and core symptoms of schizophrenia, in adult offspring following maternal immune activation [101]. Our recent study demonstrated that chronic D-serine treatment ameliorated cognitive dysfunction in a neurodevelopmental mouse model of schizophrenia. Mechanistically, we found that D-serine restores the excitation/inhibition balance by reconstituting both synaptic and intrinsic inhibitory control of cingulate pyramidal neurons. This effect was mediated through the facilitation of parvalbumin-positive (PV) interneuron-preferential NMDA receptor function and the activation of small-conductance calcium-activated potassium (SK) channels in pyramidal neurons, respectively [49]. However, it is important to note that the rapid metabolism of D-serine by the enzyme D-amino acid oxidase (DAAO) may reduce its bioavailability, potentially posing a challenge for its therapeutic use in schizophrenia [102]. Additionally, there are concerns regarding the potential nephrotoxicity associated with high concentrations of D-serine, as observed in rats developing acute tubular necrosis with higher doses [103,104]. Nonetheless, the measurement of serum D- and L-serine levels has been proposed as a potential biological marker for schizophrenia [99]. Further research with larger sample sizes and specific controls, following the guidelines for accurate measurement and detection methods [105], is warranted to fully elucidate the therapeutic potential of modulating the D-serine–NMDA receptor axis in schizophrenia.

Building upon the same lines, D-serine has been extensively investigated in numerous clinical studies, both employed alone and as an adjunct to antipsychotics, for its ability to improve the cognitive and negative symptoms of schizophrenia. Key findings from these studies are summarized in Table 2. Many clinical trials have reported significant improvements in the cognitive and negative symptoms of schizophrenia with D-serine supplementation. For instance, a pilot, double-blind, placebo-controlled, randomized parallel group, mechanistic proof-of-concept trial demonstrated a 35.7% improvement in negative symptoms (cognitive impairment is a common negative symptom of schizophrenia) compared with a placebo in individuals at high risk of schizophrenia [106]. Similarly, a study of 31 Taiwanese schizophrenic patients receiving D-serine (30 mg/kg/day) as an adjunct to standard antipsychotics revealed significant improvements in their cognition, negative and positive symptoms, as well as enhanced performance on the Wisconsin Card Sorting Test (WCST) [107]. However, not all studies have yielded positive results. A multicenter, add-on randomized controlled trial indicated that the effect of low-dose D-serine (2 g/day) on the treatment of cognitive and negative symptoms appeared to be small [108]. Conversely, the first randomized, double-blind, placebo-controlled study using a higher dose of D-serine (60 mg/kg) in schizophrenia patients reported significant improvements in clinical symptoms, suggesting that a minimum daily dose of 3.6 g of D-serine may be necessary to achieve improvements in negative symptoms [109]. It is important to note that high-dose D-serine administration can lead to adverse effects, such as peripheral neuropathies, oxidative damage [110], neurotoxicity [111], and renal toxicity [112,113].

In summary, existing clinical evidence suggests that D-serine may be a promising treatment for cognitive and negative symptoms in schizophrenia, particularly when used as an adjunct to antipsychotic medications. However, the therapeutic benefit appears to be dose dependent, and the potential for adverse effects, especially with higher doses, should be carefully considered in the design and implementation of future clinical trials.

### 4.2. Indirect Enhancement of NMDA Receptor Function

As described previously, glutamate, glycine, and D-serine directly target postsynaptic NMDA receptors and activate NMDA receptor functioning. However, the beneficial effects of directly targeting the NMDA receptor with the above compounds is limited due to several factors, including the need for high doses, a narrow therapeutic window, poor CNS penetration, and associated side effects. Alternatively, as illustrated in Figure 3, indirect enhancement of NMDA receptor function by improving the availability of synaptic glycine and D-serine levels in astrocytes provides a new approach to help meet the needs of patients with schizophrenia.

#### 4.2.1. Enhancement of NMDA Receptor Function by GlyT1 Inhibitors

Glycine transporter type 1 (GlyT1) is involved in the reuptake of glycine from the synaptic cleft. By inhibiting GlyT1, compounds such as sarcosine, BI 425809, and bitopertin can reduce the reuptake of glycine, increasing its concentration in the synaptic cleft [122]. GlyT1 is highly colocalized with NMDA receptors on glial cells and neurons in the cortex, hippocampus, septum, and thalamus [123]. GlyT1 effectively regulates synaptic glycine reuptake and enhances NMDA receptor function by promoting the binding of glycine to subtypes of NMDA receptors [124]. Thus, selective inhibition of astrocytic GlyT1 represents a promising therapeutic strategy to enhance synaptic glycine concentrations and boost NMDA receptor activity.

Multiple lines of evidence indicate that inhibition of GlyT1 enhances NMDA receptor function, which holds promise for the treatment of CIAS. BI 425809 is a novel, potent, and selective GlyT1 inhibitor that can increase synaptic glycine concentrations, thereby enhancing NMDA receptor signaling and improving neural plasticity, which in turn ameliorates cognitive function [125,126]. A randomized controlled trial found that PF-03463275 as a GlyT1 inhibitor could enhance cognitive training and neuroplasticity in schizophrenia [127]. Glycyldodecylamide, a compound that blocks neuronal glycine uptake and that may therefore increase intrasynaptic glycine levels, inhibits PCP-induced psychosis in schizophrenia [128]. Subsequent chronic (2-week) administration of (R)-(N-[3-(4′-fluorophenyl)-3-(4′-phenylphenoxy)propyl])sarcosine (NFPS, also known as ALX5407), a GlyT1 inhibitor, enhanced PCP-induced cognitive deficits [129,130]. Sarcosine, another GlyT1 inhibitor, has promising therapeutic potential in ameliorating cognitive deficits in both animal models [131,132,133] and patients with schizophrenia [134]. Intriguingly, it has been proven that sarcosine may enhance NMDA receptor function by more than one mechanism and may have different effects from NMDA receptor agonists like glycine [135]. In addition, other GlyT1 inhibitors, such as SSR103800 and SSR504734, have also exhibited similar beneficial effects in sensorimotor gating, learning and memory, and schizophrenia-like behaviors [136,137]. Furthermore, selective genetic disruption of GlyT1 resulted in the enhancement of NMDA receptor function, memory retention, and protected against amphetamine disruption of sensory gating, suggesting that inhibition of GlyT1 might have both cognitive-enhancing and antipsychotic effects [138]. These findings indicate that GlyT1 is a promising drug target for the treatment of schizophrenia-related behaviors and cognitive deficits, even though the high binding affinity of the GlyT1 inhibitor can cause unpredictable toxicity [139,140,141].

#### 4.2.2. Enhancement of NMDA Receptor Functions by DAAO Inhibitors

In contrast to GlyT1 inhibitors, another promising therapeutic strategy for treating schizophrenia is to indirectly increase synaptic D-serine levels by targeting D-amino acid oxidase (DAAO). DAAO is an enzyme that degrades D-serine. Inhibiting DAAO via DAAO inhibitors can lead to increased synaptic D-serine levels and the regulation of NMDA receptor-evoked electrophysiological activity, thereby ameliorating NMDA receptor hypofunction and the cognitive deficits observed in schizophrenia. Interestingly, the expression and activity of DAAO are found to be elevated in individuals with schizophrenia, and this enhanced DAAO activity is thought to contribute to the reduced D-serine levels and subsequent impairment in NMDA receptor functioning [102,142]. Furthermore, genetic variations in DAAO and its activator have been associated with the negative symptoms and cognitive deficits observed in schizophrenic patients [143,144,145]. Adding to the therapeutic potential of DAAO inhibition, it has been reported that certain antipsychotic medications, such as chlorpromazine (a first-generation antipsychotic) and risperidone (a second-generation antipsychotic), may possess DAAO-inhibiting properties [146,147]. Indeed, elevated levels of D-serine have been observed in rodents after the administration of DAAO inhibitors [148,149]. Consistently, pre-pulse inhibition deficits and cognitive deficits relevant to schizophrenia were ameliorated after treatment with DAAO inhibitors [149,150]. Moreover, mutant mice lacking DAAO exhibit increased NMDA receptor function [151] that facilitated hippocampal LTP and spatial learning [152]. Other animal studies have indicated that DAAO is involved in the mechanism of D-serine nephrotoxicity [153], which also could be attenuated by DAAO inhibitors [154]. Therefore, DAAO inhibitors, combined with D-serine or used alone, might be beneficial for enhancing NMDA receptor function in schizophrenia.

#### 4.2.3. Enhancement of NMDA Receptor Function by Other NMDAR Modulators

Several compounds that modulate NMDA receptor activity indirectly have been investigated as potential therapeutics for schizophrenia. For example, agents that target the glycine site on the NMDA receptor, such as D-cycloserine (DCS), have shown mixed results in treating negative symptoms and cognitive impairment in schizophrenia [155]. Lower doses of 50 mg/day produced persistent benefits for negative symptoms and memory deficits when added to first-generation antipsychotics [156,157], but higher doses of 100 mg/day or more worsened psychotic symptoms [158]. The Cognitive and Negative Symptoms in Schizophrenia Trial (CONSIST) found no significant improvements in negative symptoms or cognition when DCS was added to second-generation antipsychotics [159]. Similarly, the efficacy of DCS did not achieve statistical significance in a meta-analysis of add-on trials, in contrast to the more consistently positive results with the full NMDA receptor co-agonist glycine [157,160]. Beyond its effects on symptoms, DCS positively modulated NMDAR-dependent forms of LTP and LTD in the hippocampal brain slices of juvenile rats without affecting basal synaptic transmission [161]. The modulation of synaptic plasticity by DCS may contribute to its potential therapeutic effects, though the clinical efficacy appears to be influenced by factors like antipsychotic medication type. Furthermore, research in rats has shown that the actions of endogenous kynurenic acid on VTA dopamine (DA) neurons are antagonized by DCS [162]. This suggests that elevated levels of kynurenic acid impact the basal firing characteristics of VTA DA neurons, specifically by blocking the glycine site of the NMDA receptor. Collectively, these findings highlight the interactions between DCS, NMDA receptor modulation, and the treatment of cognitive and negative symptoms in schizophrenia.

Other modulators, including allosteric modulators and subtype-selective agonists, are also being explored for their therapeutic potential. These modulators bind to sites distinct from agonist binding sites, inducing conformational changes that enhance or inhibit receptor function. Positive allosteric modulators (PAMs) can enhance NMDA receptor activity, potentially offering benefits for cognitive enhancement and treating psychiatric disorders. Relatively non-selective PAMs that alter NMDA receptor function independent of subunit composition, such as CAD-9303, have been studied as treatments for the cognitive deficits and negative symptoms of schizophrenia [21]. GluN2A-selective PAMs, including GNE-6901 and GNE-8324, have provided a proof-of-principle for the development of allosteric modulators of NMDA receptors; however, their poor pharmacokinetic properties and poor central nervous system exposures hinder their uses in vivo [163]. These PAMs also showed cell-selective functional differences in brain slice neurophysiology experiments, with GNE-6901 enhancing NMDA receptor synaptic responses on both excitatory and inhibitory neurons, whereas GNE-8324 selectively enhanced NMDA receptor responses on inhibitory neurons but not excitatory neurons. The reason for this synaptic selectivity might involve differences in the microenvironment between synapses on excitatory and inhibitory neurons that result in different susceptibilities to potentiation by specific modes of PAM action [70]. Accumulating evidence suggests that GluN2B PAMs may have effects on cognitive function [70,164]. In contrast, negative allosteric modulators (NAMs) of GluN2B have been shown to induce transient cognitive impairment [165] similar to the actions of NMDA receptor blockers, suggesting that GluN2B potentiation may produce an opposing effect.

Overall, targeting NMDA receptors presents a logical approach to addressing the underlying neurobiological abnormalities in schizophrenia. By restoring NMDA receptor function and enhancing synaptic plasticity, these therapeutic strategies have the potential to improve cognitive deficits and alleviate symptoms, thereby enhancing the quality of life for individuals with schizophrenia.

## 5. Conclusions

The NMDA receptor hypofunction hypothesis provides a framework for understanding the neurobiological basis of schizophrenia, particularly its cognitive impairment. Targeting NMDA receptor pathways represents a promising avenue for developing novel therapeutics aimed at restoring cognitive function and improving outcomes for individuals affected by schizophrenia. Data from clinical and animal studies have strongly implicated NMDA receptors as central hubs in the complex pathophysiological processes underlying schizophrenia. Accordingly, therapeutic drugs for CIAS that are based on the regulation of NMDA receptor function are currently under development. Importantly, several NMDAR-enhancing agents, particularly those that indirectly modulate NMDA receptor function, have demonstrated significant alleviation in schizophrenia-like behavioral deficits and cognitive dysfunctions in both animal models and patients with schizophrenia. Moreover, current findings suggest that indirectly targeting NMDA receptors appears to be more beneficial and results in fewer adverse effects than directly modulating NMDA receptor function. Additionally, as the development of new antipsychotic drugs progresses, the establishment of comprehensive safety profiles for these potential compounds will be highly informative. This could elucidate their precise mechanisms of action and enable the evaluation of their therapeutic effects in both animal models and clinical studies.

Notably, however, this review offers an oversimplified summary of treatment alternatives for a highly complex psychiatric disorder. The functioning of the glutamate system, particularly NMDA receptors, is influenced by sex differences [166]. Understanding these differences is crucial, especially regarding their implications for cognitive impairment and negative symptoms in schizophrenia. Clinical trials are essential in drug development, while preclinical animal studies provide valuable insights into underlying mechanisms and help inform new therapeutic interventions. Lumateperone (also known as ITI-007 or ITI-722) has been shown to be effective in treating the positive, negative, and cognitive symptoms of schizophrenia [167]. It simultaneously modulates key neurotransmitters, including serotonin, dopamine, and glutamate [168]. Future research that integrates single-cell sequencing techniques may uncover cell-type-specific NMDA receptor hypofunction in schizophrenia. This could help identify potential biomarkers for multi-target drugs like lumateperone, leading to more precise and personalized clinical treatments. It is important to note that not all patients with schizophrenia experience cognitive deficits or primary negative symptoms, highlighting the disorder’s heterogeneity. Advances in cognitive assessment, such as the MATRICS Consensus Cognitive Battery (MCCB), enable a comprehensive evaluation of cognitive functioning across various domains. This recognition of individual cognitive profiles is vital for personalizing treatment strategies and improving outcomes, including those targeting glutamatergic systems.

## Figures and Tables

**Table 1:** Effects of glycine on the treatment of schizophrenic symptoms in clinical studies.

Compound	Sample Size (Placebo vs. Experiment)	Dosage	Clinical Results	References
Glycine	11 (no placebo)	5–25 (g/day)	No significant effects on symptoms	[78]
	6 (no placebo)	10.8 (g/day)	No significant effects on symptoms	[79]
	6 (no placebo)	15 (g/day)	No significant effects on symptoms	[80]
	7 vs. 7	2–30 (g/day)	Significant improvements in negative symptoms	[81]
	5 (no placebo)	0.14–0.8 (g/kg/day)	Significant improvements in negative symptoms	[82]
	11 vs. 11	0.8 (g/kg/day)	Significant improvements in depressive, cognitive, and negative symptoms	[83]
	22 vs. 22	0.8 (g/kg/day)	Significant improvements in depressive, cognitive, and negative symptoms	[84]
	10 vs. 9	30 (g/day)	No significant effects on symptoms	[85]
	13 vs. 14	60 (g/day)	No significant effects on symptoms	[86]
	6 vs. 6	0.2–0.8 (g/kg/day)	Significant improvements in negative symptoms	[87]
	12 vs. 12	60 (g/day)	No significant effects on symptoms	[88]
	2 vs. 2	6–48 (g/day)	Significant improvements in clinical symptoms	[89]
	2 (no placebo)	5.4–86.5 (g/day)	Significant improvements in clinical symptoms	[90]
	10 vs. 10	0.8 (g/kg/day)	No significant effects on symptoms	[91]
	29 (no placebo)	0.8 (g/kg/day)	Significant improvements in positive and negative symptoms	[92]

**Table 2:** Effects of D-serine on the treatment of schizophrenic symptoms in clinical studies.

Compound	Sample Size (Placebo vs. Experiment)	Dosage	Clinical Results	References
D-serine	15 vs. 14	30 (mg/kg/day)	Significant improvements in cognitive, negative, and positive symptoms	[107]
	10 vs. 10	30 (mg/kg/day)	No significant effects on symptoms	[114]
	38 vs. 37	20–30 (mg/kg/day)	Significant improvements in negative, positive, cognitive, and depression symptoms	[115]
	16 (20) vs. 16 (20)	2 (g/day)	No significant effects on symptoms	[116]
	12 vs. 19 vs. 16	30, 60, 120 (mg/kg/day)	Significant improvements in symptoms	[113]
	69 (98) vs. 73 (97)	2 (g/day)	No significant effects on symptoms	[108]
	5 (10) vs. 3 (8)	1.5–3 (g/day)	Significant improvements in negative symptoms	[117]
	23 (26) vs. 25 (27)	30 (mg/kg/day)	No significant effects on symptoms	[118]
	17	1.5–4 (g/day)	Significant improvements in symptoms	[119]
	20 (24) vs. 15 (20)	60 (mg/kg/day)	Significant improvements in negative symptoms	[106]
	13	60 (mg/kg/day)	Improvements in auditory plasticity	[120]
	16 vs. 16	60 (mg/kg/day)	Significant improvements in symptoms	[109]
	9 vs. 12	100 mg/kg	Significant plasticity improvements	[121]
